# Supplementary material for: Changes in Essential Oil Composition, Polyphenolic Compounds and Antioxidant Capacity of Ajowan (Trachyspermum ammi L.) Populations in Response to Water Deficit
Source: Foods. 2022 Oct 5;11(19):3084. doi: 10.3390/foods11193084 (PMC9562171; doi:10.3390/foods11193084)
Supplement: Supplementary file 1 [file foods-11-03084-s001.zip › foods-1923978-supplementary.pdf]

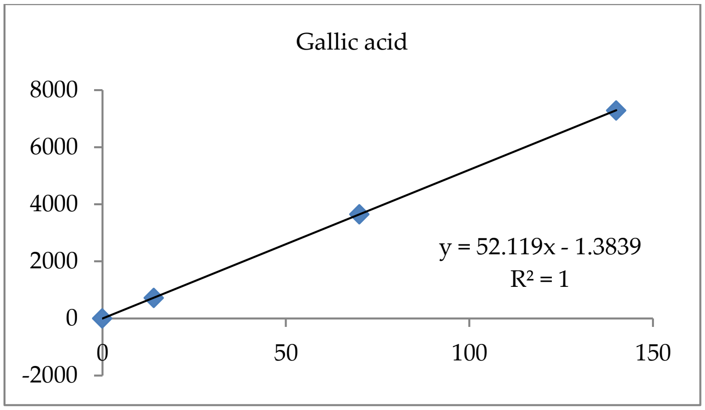
Gallic acid
8000
6000
4000
2000
0
-2000
0
50
100
150
y = 52.119x - 1.3839
R² = 1

(a)

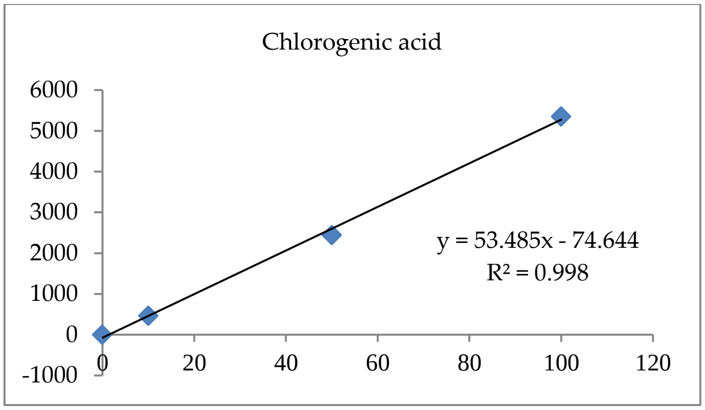
Chlorogenic acid
6000
5000
4000
3000
2000
1000
0
-1000
0
20
40
60
80
100
120
y = 53.485x - 74.644
R² = 0.998

(b)

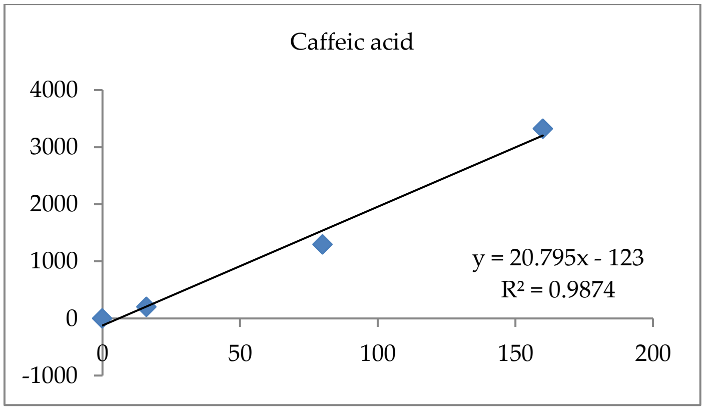
Caffeic acid
4000
3000
2000
1000
0
-1000
0
50
100
150
200
y = 20.795x - 123
R² = 0.9874

(c)

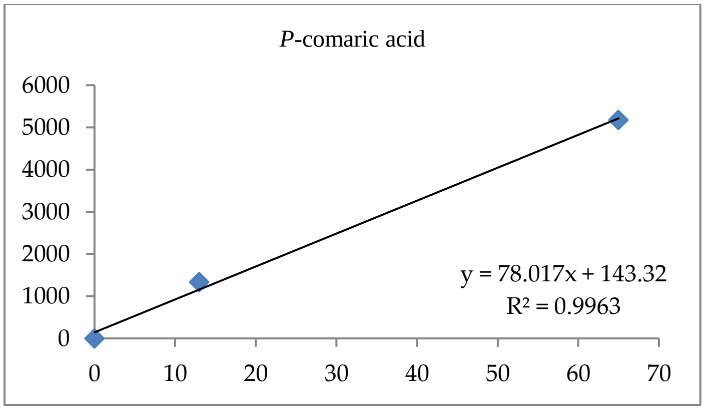
P-comaric acid
6000
5000
4000
3000
2000
1000
0
0
10
20
30
40
50
60
70
y = 78.017x + 143.32
R² = 0.9963

(d)

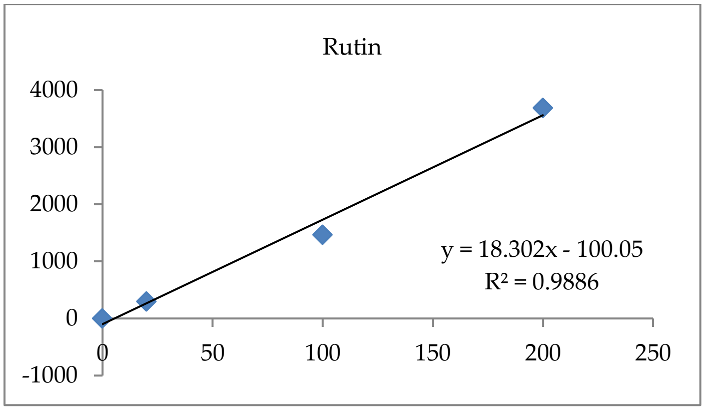
Rutin
4000
3000
2000
1000
0
-1000
0
50
100
150
200
250
y = 18.302x - 100.05
R² = 0.9886

(e)

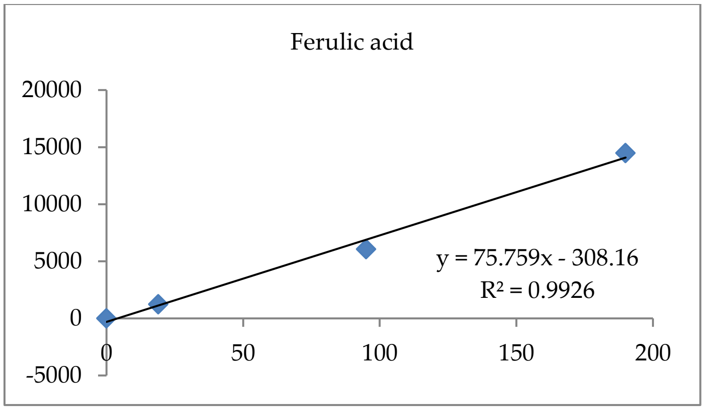
Ferulic acid
20000
15000
10000
5000
0
-5000
0
50
100
150
200
y = 75.759x - 308.16
R² = 0.9926

(f)

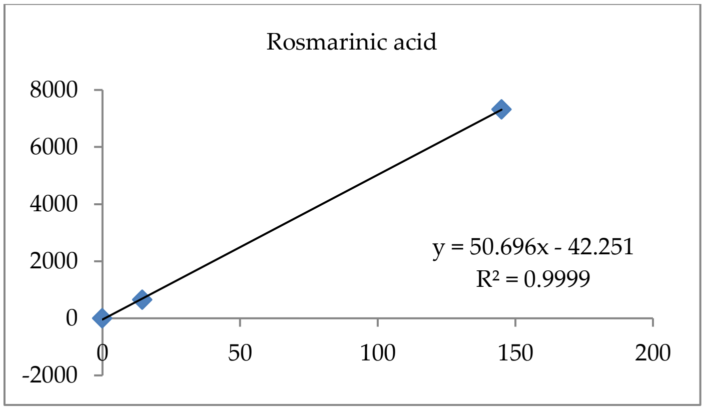
Rosmarinic acid
8000
6000
4000
2000
0
-2000
0
50
100
150
200
y = 50.696x - 42.251
R² = 0.9999

(g)

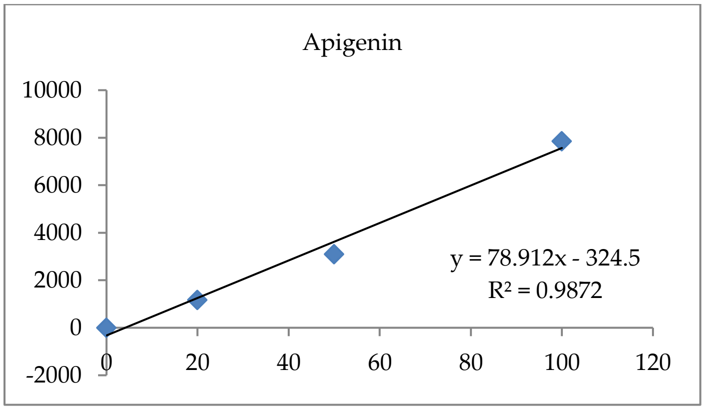
Apigenin
10000
8000
6000
4000
2000
0
-2000
0
20
40
60
80
100
120
y = 78.912x - 324.5
R² = 0.9872

(h)

Fig 1S. Calibration curves of polyphenolic standards a) Gallic acid, b) Chlorogenic acid, c) Caffeic acid, d) *p*-coumaric acid, e) Rutin, f) Ferulic acid, g) Rosmarinic acid, h) Apigenin.
